# Does an Eye-Hand Coordination Test Have Added Value as Part of Talent Identification in Table Tennis? A Validity and Reproducibility Study

**DOI:** 10.1371/journal.pone.0085657

**Published:** 2014-01-17

**Authors:** Irene R. Faber, Frits G. J. Oosterveld, Maria W. G. Nijhuis-Van der Sanden

**Affiliations:** 1 Faculty of Physical Activity and Health, Saxion University of Applied Sciences, Enschede, The Netherlands; 2 Scientific Institute for Quality of Healthcare, Radboud University Medical Centre, Nijmegen, The Netherlands; Universidad Europea de Madrid, Spain

## Abstract

This study investigated the added value, i.e. discriminative and concurrent validity and reproducibility, of an eye-hand coordination test relevant to table tennis as part of talent identification. Forty-three table tennis players (7–12 years) from national (n = 13), regional (n = 11) and local training centres (n = 19) participated. During the eye-hand coordination test, children needed to throw a ball against a vertical positioned table tennis table with one hand and to catch the ball correctly with the other hand as frequently as possible in 30 seconds. Four different test versions were assessed varying the distance to the table (1 or 2 meter) and using a tennis or table tennis ball. ‘Within session’ reproducibility was estimated for the two attempts of the initial tests and ten youngsters were retested after 4 weeks to estimate ‘between sessions’ reproducibility. Validity analyses using age as covariate showed that players from the national and regional centres scored significantly higher than players from the local centre in all test versions (*p*<0.05). The tests at 1 meter demonstrated better discriminative ability than those at 2 meter. While all tests but one had a positive significant association with competition outcome, which were corrected for age influences, the version with a table tennis ball at 1 meter showed the highest association (r = 0.54; *p* = 0.001). Differences between the first and second attempts were comparable for all test versions (between −8 and +7 repetitions) with ICC's ranging from 0.72 to 0.87. The smallest differences were found for the test with a table tennis ball at 1 meter (between −3 and +3 repetitions). Best test version as part of talent identification appears to be the version with a table tennis ball at 1 meter regarding the psychometric characteristics evaluated. Longitudinal studies are necessary to evaluate the predictive value of this test.

## Introduction

Table tennis is widely judged as one of the fastest sports, and can be described as a fairly difficult task. It is an open, complex motor task that requires performance in a constantly changing environment under great time pressure [1], [2], [3]. High performance in table tennis requires a broad repertoire of movements allowing quick and responsive adaptation to the continuously changing conditions [3], [4]. Players need to develop outstanding technical skills, fast switching capability to adjust stroke techniques, variable, flexible and fast footwork, pronounced ability to react and anticipate, proper positioning, and balance control [5]–[8]. Moreover, also highly developed tactical skills, decision-making ability, creativity, concentration, competitiveness, apprehension, self-regulation, and willpower are inseparable to excel in this sport [9]–[12]. Although it is not the only aspect, table tennis appeals significantly on a player's coordinative ability or motor skills [2], [13], [14], [15].

Talent identification programs in table tennis ought to find gifted youngsters at an early stage to educate them to top players effectively and efficiently [16], [17], [18]. Children who are gifted with outstanding natural abilities in the sensorimotor domain are suggested to be in advantage to other children by an easier and faster learning process for this type of sport [19], [20]. Such an effective and efficient learning or performance curve might be a predictor of the child's full potential [21], [22]. The learning process in table tennis will be facilitated further starting at a young age (6–12 years) by using the most sensitive period to develop motor skills [13], [23], [24]. Consequently, assessing natural motor abilities necessary for table tennis in youngsters (6–12 years) beside cognitive and social-emotional abilities as part of talent identification seems reasonable to identify high potentials for this sport. However, the question arises how to measure this giftedness?

Motor tests with proven predictive value for talent identification in table tennis or other (racquet) sports are lacking [19], [25]. Vandorpe et al. (2012) was the first to show that general motor coordination tests correlate strongly with competition results in elite gymnast; the tests explained more than 40% of the variation in competition performance two years later [26]. The authors suggested that the tests measure a general trait underlying a wide variety of skills and that they are sensitive enough to give an indication of potential performance. Probably the measurement of this underlying trait reflects an essential natural ability for gymnastics [26]. This is also in accordance with the idea that to measure future potential one should not assess specific sport skills themselves in order to limit the influence of training-experience [18], [20], [27]. Although the study of Vandorpe et al. (2012) was conducted in gymnasts (7–8 years), the concept of the study is believed to suit table tennis, because both sports require an ‘early specialisation’ to develop complex motor skills. While for gymnastics dynamic balance seemed the most important predictor for potential, in table tennis eye hand coordination necessary for ball control, speed and rapidity to react on environmental changes, agility, and reaction and anticipation would probably be more adequate predictors [5]–[8].

The Netherlands Table Tennis Association (NTTA) is developing a talent identification assessment (TIDA) to identify gifted table tennis players in the age range of 6–12 years. This TIDA includes seven test items for assessing eye hand coordination, ball control, coordination, speed/rapidity, anaerobic power and agility [28]. Although these test items already seem to measure all underlying motor abilities, the trainers and coaches were not completely satisfied with the assessment regarding eye hand coordination. Two items specifically intend to measure a combination of eye hand coordination, ball control and anticipatory skills by using an aiming-task under different circumstances. However, these test items assess self-induced discrete movements and do not require repetitive movements with the necessity to anticipate and react quickly under time pressure, which is demonstrated to be essential in table tennis [5], [6], [29], [30].

In cooperation with trainers and coaches of the NTTA, a new test item was developed to measure eye-hand coordination and ball control under time pressure [31]. This test item included a non-specific table tennis task with serial movements requiring continuously adapted responses. The new test item was developed for players from 6–12 years of age and practical feasibility was taken into account [13], [27]. After several tryouts and discussion rounds, four different test versions were proposed. Since it was not clear which test version would fit best for talent identification, all versions were investigated on discriminative and concurrent validity, and reproducibility in this study. It was hypothesized that the test could discriminate players from apparent different training levels. Moreover, positive moderate significant associations (r between 0.4 and 0.7) between test and performance results (concurrent validity) were expected in young table tennis players. Reproducibility was hypothesized to be at an acceptable level due to the inclusion of a training-phase, a time-constraint repetitive-performance task and two attempts [27]. Eventually, only the test version with the best feasibility regarding above-mentioned aspects will be selected for implementation in the TIDA of the NTTA as part of talent identification. The selected test version together with the other test items will be put under further investigation about their value for talent identification in future studies. This study contributes to an evidence-based TIDA for table tennis, which supports better talent identification in table tennis.

## Methods

### Ethics statement

The study protocol and informed consent procedure were approved by the Ethics Committee of the Medical Spectrum Twente (Medical School Twente, Institute for Applied Science, Enschede, the Netherlands; MTC/12307.fab 2-10-2012) in full compliance with the Declaration of Helsinki. Written informed parental consent and player assent were obtained. Furthermore, both the children and their parents have given written informed consent, as outlined in the PLOS consent form, to publication of the video clips.

### Study design

To investigate validity the design was two-fold. First, all four test versions of the eye hand coordination test were evaluated, using the so-called ‘known group method’ [32], on their expected ability to discriminate between young table tennis players from national, regional and local training centres. Secondly, the associations between the results of the four test versions and competition outcome were examined for concurrent validity. Furthermore, this investigation used the initial test including two attempts and a test–retest research design to examine the ‘within session’ and the ‘between sessions’ reproducibility, respectively. The time between the initial test and retest was four weeks.

### Participants

Young players were recruited from the national and a regional training centre of the NTTA and two local table tennis centres. Players at the national training centre were selected by expert trainers of the NTTA and were suggested to be the most gifted players at that moment throughout the Netherlands. The players of the regional training were selected by the NTTA trainers of the eastern department and were considered to be the most gifted players of that region. The children recruited from the local centre were judged by their trainers as not competent for a regional and/or the national training centre of the NTTA at that moment and also in future. Inclusion criteria were: an age between 6–12 years and being a member of a table tennis club connected to the NTTA. Players with injuries were excluded. Written informed parental consent and player assent were obtained prior to testing.

### Measurements

**Figure 1:**
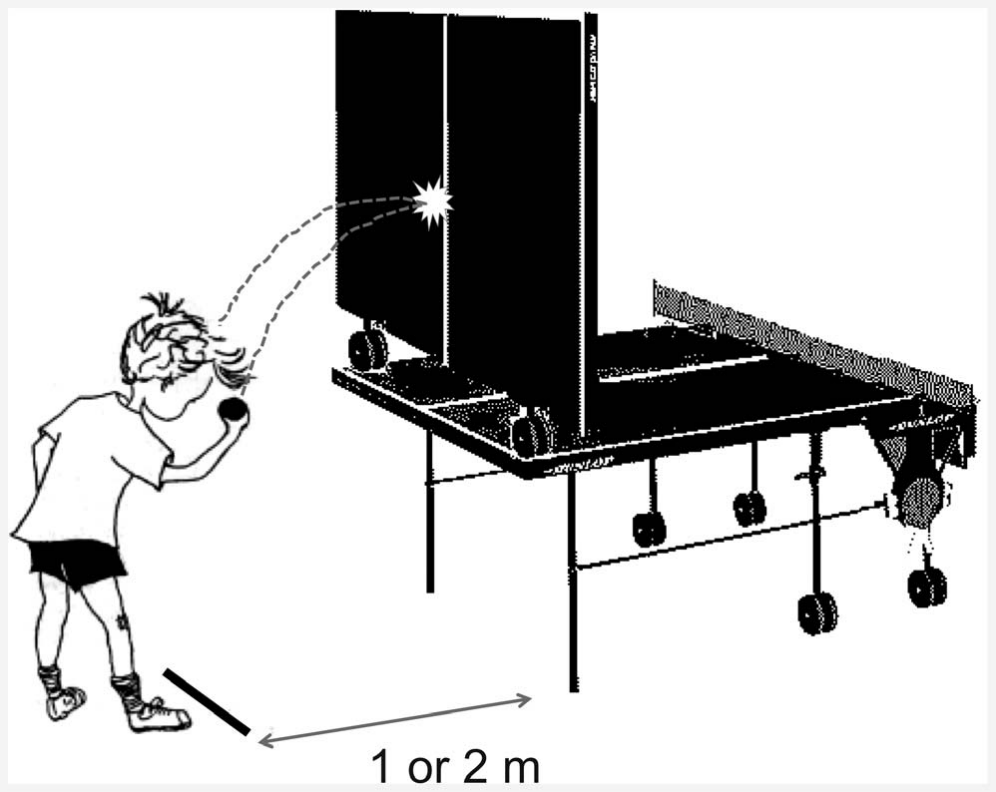
Eye-hand coordination test set-up.

Standardization of the test was realized in a protocol that included a detailed description for materials, set-up, assignment, demonstration, training-phase, testing-phase and registering test-scores [31]. Players were instructed to throw a ball to a vertical positioned table tennis table with one hand and to catch the ball correctly with the other hand as frequently as possible in 30 seconds (Figure 1; Video S1, Video S2, Video S3, Video S4). The players were free to use overhand (Video S3, Video S4) and/or underhand techniques (Video S1, Video S2) or a combination of both for throwing and catching. Consequently, players were able to use their best motor performance strategies for optimal results, which is analogous with the table tennis context. The test consisted of four test versions in which the distance to the table tennis table (1 or 2 meter) and the type of ball (table tennis or tennis ball) were varied. All test versions included a training-phase and a testing-phase. Players were familiarized with the test during the training-phase; they practiced throwing and catching of the ball six times only before the first attempt. Procedural inaccuracies were corrected during this phase. In the testing-phase, the highest number correctly caught balls in 30 seconds of two attempts was registered as final outcome. No feedback was given during the testing-phase. The total time for testing was about 5–10 minutes per player.

### Procedure

All players were assessed under similar conditions at a regular training at their training centre on a Sunday morning. Before testing all participants did a warming-up as part of their training. After warming-up children started with the specific table tennis training and were invited for the eye-hand coordination test one by one. The order of the four different test versions was randomised for the players of all training centres using playing cards. After the test, the youngsters returned to their training. Testers were four physiotherapy students who were trained to the same extent in using the test protocols. They were familiarized with the test-protocol and instruction and feedback was given during training by an expert-trainer of the NTTA. Ten players from the same regional training centre were recruited for retesting after four weeks to estimate reproducibility. The retest was conducted by a different tester, because this best matches daily practice where different trainers conduct the motor tests [33]. The tester of the retest was blinded for the test outcome of the initial test. Competition results (points) were provided by the NTTA to investigate the relationship between test outcomes and table tennis performance. A higher score in competition points was considered as a better performance.

### Statistical analysis

PASW Statistics 21 for Windows (SPSS, Inc., Chicago, Illinois, United States of America) was used for the statistical analyses. Normality of test outcomes was evaluated by the Shapiro-Wilk test. Differences in group characteristics were tested using a one-way ANOVA and Bonferroni post-hoc tests for ratio type data, and a chi-square test for frequencies.

Then first, ANCOVA and Sidek post-hoc tests were used to test whether the four test versions discriminated between the players from the national, regional and local training centres. Test outcomes of the four test versions were inserted one by one as dependent variables, the training centres as fixed factor, and age and training hours as suggested covariates. Secondly, the relationships between the test scores of all versions and the competition outcome were evaluated using partial correlation coefficients intending to control for age and training hours. Finally, the ‘within session’ and ‘between sessions’ reproducibility were analysed. Bland-Altman plots were used for each test version to provide a visual representations of measurement errors against true values by plotting a) the difference between the values of the two attempts of the initial test versus the mean of these two attempts and b) the difference between the initial test and the retest values versus the mean of initial test and retest scores [34]. Additionally, intraclass correlation coefficients (ICC) were calculated for the ‘within session’ reproducibility based on the one-way random model. For the ‘between sessions’ reproducibility only Bland-Altman analyses were selected, due to the relatively small and homogenous sample that was used for the retest. Alpha was set on 0.05 for significance for all analysis.

## Results

In total 43 young Dutch table tennis players (age 7–12 years) from the national, regional and local training centres participated in this study. This included all players of the national training centre of the NTTA (n = 13) and all players of the regional training centre of the eastern department (n = 11) at that time. From the players recruited at the local club (n = 19), six players did not participate in an official competition of the NTTA. Characteristics of the participants are presented in Table 1. No significant differences were found between the three groups regarding gender, age, height, weight and handedness. Yet, training (*p*<0.001) and competition results (*p*<0.001) differed significantly between groups.

**Table 1:** Characteristics of participants.

	Total	National	Regional	Local
Total	43	13	11	19
Boys	26	8	8	10
Girls	17	5	3	9
Age (years)	10.4±1.4	10.9±1.5	10.4±1.5	10.1±1.4
7 year olds	1	-	-	1
8 year olds	5	1	1	3
9 year olds	3	-	3	-
10 year olds	12	3	2	7
11 year olds	11	5	1	5
12 year olds	11	4	4	3
Length (cm)	149±11	150±12	150±12	148±10
Weight (kg )	38±8	37±7	37±7	38±9
Right-handed	35	9	9	17
Left-handed	8	4	2	2
Training (hours*week-1)	6 (0–20)	11 (7–20)	7 (4–11)	2 (0–3)
Competition (points)	173 (−52–430)	297 (144–430)	188 (72–317)	36 (−52–130)

Data are frequencies, except for age, length and weight (years±SD), and training and competition (mean (range)).

Outcomes of each group on all test versions were normally distributed; *p*-values of the Shapiro-Wilk tests were >0.05. The results of the validity analyses are presented in Table 2. Participants tended to have higher scores on the test versions at 1 meter distance compared to the versions at 2 meter, and also on the test versions with the tennis ball compared to the versions with the table tennis ball. Most children used only underhand techniques for throwing the ball at the test versions. Only five children used a combination of overhand and under hand techniques (national n = 1, regional n = 3, local n = 1). All four test versions showed a significant difference between the players of the national and the local training centres (*p*<0.05) and between the players of the regional and local training centres (*p*<0.05). However, the differences between the players of the national and the regional training centre were not significant for all test versions. These results are based on an ANCOVA using only age as a covariate. Training hours appeared to be a characteristic of the different training centres and not an independent covariate. Influences of training hours can therefore not be estimated correctly in this analysis. A similar problem appeared at the calculation of the partial correlations; they could not be controlled correctly for training hours because of high collinearity with the competition outcomes. Only controlling for age, the results from all test versions but one were significant and positively associated with the competition results (*p*<0.05). The partial correlation coefficient of the test version at 2 meter distance with a tennis ball did not reach the level of significance. The tests version at 1 meter distance with a table tennis ball showed the strongest association (r = 0.54; *p* = 0.001).

**Table 2:** Results of eye hand coordination by test version (correctly caught balls/30seconds; mean±SD).

Test version	National	Regional	Local	F (*p*)	r (*p*)
	(*n* = 13)	(*n* = 11)	(*n* = 19)	(*n* = 43)	(*n* = 37)
Tennis ball - 1 m	29±5	26±5	17±7	15.722* (<0.001)	0.51* ( = 0.002)
Tennis ball - 2 m	17±5	18±3	12±5	6.616* ( = 0.003)	0.17 ( = 0.316)
Table tennis ball - 1 m	24±7	23±4	14±5	18.507* (<0.001)	0.54* ( = 0.001)
Table tennis ball - 2 m	15±6	16±4	9±5	9.747* (<0.001)	0.33* ( = 0.049)

r: Partial correlation coefficient of association between test and competition results controlled for age.

*p*<0.05: Sidak post hoc tests showed significant differences between (1) national and local level and (2) regional and local level for all test versions.

**Figure 2:**
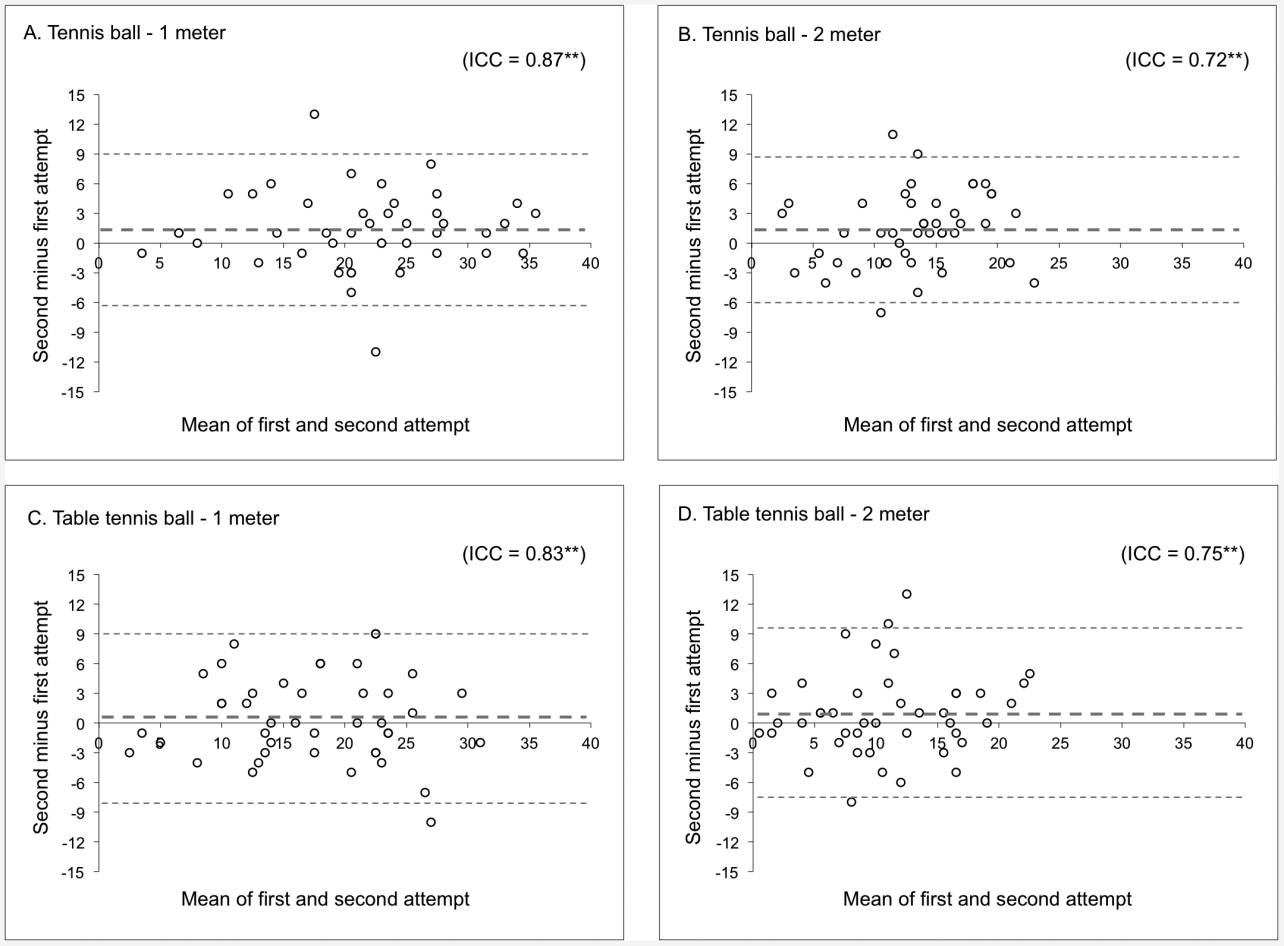
Bland-Altman plots for ‘within session’ reproducibility; A. Tennis ball - 1 meter, B. Tennis ball – 2 meter, C. Table tennis ball – 1 meter, D. Table tennis ball – 2 meter. The bold dotted line represents the mean difference (number of correctly caught balls/30 seconds) between the first attempt and second attempt. The non-bold dotted lines represent the 95% limits of agreement (±1.96•SD). ICC: intraclass correlation coefficients; ** *p*<0.01.

**Figure 3:**
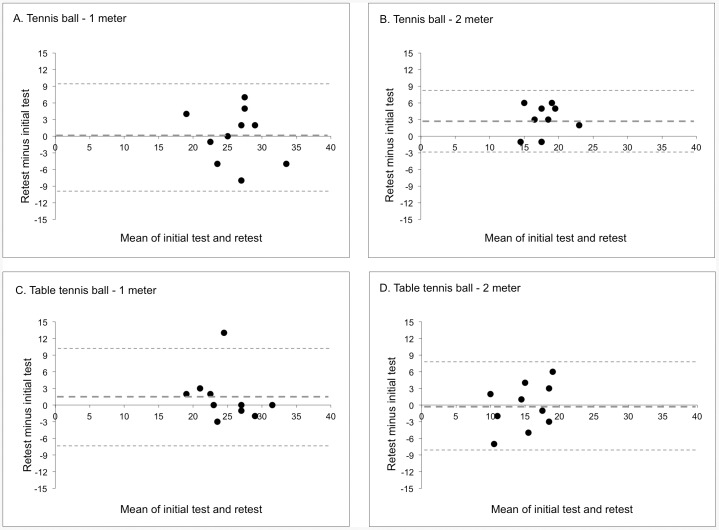
Bland-Altman plots for ‘between session’ reproducibility; A. Tennis ball - 1 meter, B. Tennis ball – 2 meter, C. Table tennis ball – 1 meter, D. Table tennis ball – 2 meter. The bold dotted line represents the mean difference (number of correctly caught balls/30 seconds) between the initial test and retest. The non-bold dotted lines represent the 95% limits of agreement (±1.96•SD).

Figure 2 A, B, C, D and Figure 3 A, B, C, D present the Bland-Altman plots for all test versions for the ‘within session’ and ‘between session’ reproducibility, respectively. The mean difference or systematic error of the ‘within session’ analysis were between 0 and 1 correctly caught balls in 30 seconds for all test items (Figure 2 A, B, C, D). The 95% confidence intervals for the difference between the first and second attempt were comparable for the four test versions; between −8 to +9 correctly caught balls in 30 seconds (Figure 2 A, B, C, D). ICC's calculated for the ‘within session’ reproducibility were significant (*p*<0.01) and ranged from 0.72 to 0.87.

In general the systematic error in all tests was also small in the ‘between session’ results (between −1 and +1 correctly caught balls in 30 seconds; Figure 3 A, C, D). Only the test version with a tennis ball at 2 meter distance presented a larger systematic error between the initial test and retest (mean difference  = +3; Figure 3 B). The test version with a table tennis ball at 1 meter distance demonstrated the smallest bandwidth on the difference between the test and retest of −3 and +3 correctly caught balls in 30 seconds for 90% of the players retested (Figure 3 C). There seemed to be one outlier (+13). The other test versions had larger differences between the initial test and retest; tennis ball 1 meter between −8 and 7 (Figure 3 A), tennis ball 2 meter between −1 and 6 (Figure 3 B), table tennis ball 2 meter between −7 and 6 (Figure 3 D).

## Discussion

The results of this study confirmed that all test items were able to discriminate local training centre players from players of the regional and national training centres, and we found positive moderate significant associations with competition results for three test versions. These main effects included a correction for the influence of age. Moreover, reproducibility was acceptable. The test version with a table tennis ball at 1 meter distance is suggested to fit best in the provisional TIDA of the NTTA as part of talent identification in table tennis, due to the best combination of psychometric characteristics examined in this study. This selected test version together with the other test items of the TIDA must be evaluated further on their predictive value in a longitudinal study including analyses which investigate the main and interaction effect of age.

It is suggested that the better validity and reproducibility results of the test version with a table tennis ball at 1 meter distance is due to the difficulty of that particular test version. Taking the mean values of the four test versions into account for all groups (Table 2), it appears that shortening the distance to the vertical positioned table tennis table improves the discriminative ability by generally enlarging the difference between groups. Due to the shorter distance the load on reaction is higher, and the youngsters had to be most precise and quick in their reactions. Table tennis players with high potential are assumed to be more prone to especially these specific quick reaction tasks than those with low potential [5], [6], [20]. The demonstrated larger difference between the players of the local training centres and the players of the national training centre in the test versions using the short distance (1 meter) compared to the large distance (2 meter) is considered logical. The ball type, on the other hand, did not influence the discriminative ability between groups in this sample. All groups had lower scores catching the table tennis ball compared to the tennis ball, which can be explained by the extra need for precision using a smaller, lighter, harder and smoother ball [1], [2], [3]. Still, the differences between the three player groups were not larger using a specific ball.

As shown all test versions had the ability to discriminate between the players of the local and other training centres. However, there was no significant difference between the players from the national and the regional training centres in this study. This could most likely be explained that both centres include children with good to excellent motor performance. This is supported by the large overlap between the competition results of both groups (Table 1). Probably, differentiating between players of regional and national training centres is more difficult regarding motor performance than between players of these centres and players of the local training centre. This could be due to other selection criteria for the trainings centres than motor performance like motivation, concentration and self-regulation, and contextual factors like the influence of parents.

This also brings up the question to what extent the test results were influenced by the difference in training experience of the players of the included training centres. Unfortunately, the influence of training could not be estimated in this cross-sectional study, due to the dependency and collinearity of intended covariate ‘training hours’ with the test groups and the competition results, respectively. Yet, the influence of training was not regarded as a subject in this study. Longitudinal studies are needed to find answers on this matter. It is believed that motor tests can only partly explain future performance, probably by reflecting a preposition or necessity for a certain skill. Deliberate practice is also considered as an essential component in talent development [19]. One of the main goals of this study was to investigate the discriminative validity of the test versions, and for that purpose a ‘known-group’ design was chosen [32]. It was the intention to examine players with a known difference in potential. Thus, players were deliberately recruited from different trainings centres consequently including unavoidable differences in training hours. By selecting a motor task which assesses the eye hand coordination which is close to table tennis, but not a specific task which is practiced during training, it was intended to measure potential and eliminate the effect of training or competition experience. Task specificity theories and studies support this consideration [35], [36]. Anyway, the main result of this study is that the test item is able to discriminate between high and low performers and all factors affecting this difference, which is an essential quality of a test for talent identification. Future studies must reveal the influence of training on test results used as part of talent identification.

Probably also due to the included sample with sufficient low and high potentials, the test results of the versions at 1 meter distance had a moderate significant relationship with the competition outcome as expected. Players from the national training centre were generally thought to be high performers and high potentials and players from the local training centre were generally considered as low performers with low potential. Yet, a strong relationship between test results and ranking was not hypothesized for this sample. Youngsters that have just started do not have that much experience and probably train at the local centres. Consequently, gifted children with little experience in competition have probably low ranking but could be high potentials for table tennis and have outstanding results at the test. And vice versa, less gifted children with a lot of training and competition experience can have a high ranking but disappointing results at the test.

‘Within session’ and ‘between sessions’ reproducibility outcomes were satisfactory. Systematic errors of all test versions in both the ‘within session’ and ‘between session’ estimations were small for all test versions, meaning no substantial learning-effects exist. The random errors shown in the ‘within session’ analyses were quite large. By using the best of two attempts as an outcome of the test versions, this random errors seem to be somewhat reduced by demonstrating a smaller random error in the ‘between session’ analyses. The selected test version for the NTTA's TIDA with a table tennis ball at 1 meter distance demonstrated the smallest random measurement error between the initial test and the retest. The outlier at the reproducibility analysis (scoring 13 balls more at the retest than at the initial test (figure 3 C) could, according to the trainer, be explained by motivational problems during the initial test. This was also seen at the other test versions, however to a lesser extent; the player caught between 2 to 5 balls more at the retest. The rather high reliability parameters (ICC's) of the ‘within session’ estimations suggest that the players can be distinguished quite well despite measurement errors. When ICC's could be calculated in a larger sample for the ‘between sessions’ reliability these are expected to show even higher values as a results of a smaller random error [37].

Then, because the protocol did not prescribe a fixed way how to perform the test both overhand as underhand techniques were used for throwing and catching. Most children (n = 38) chose to use only underhand techniques, so is not expected that this influenced the results of this study to a large extend. Remarkable was that especially children with high performances on the test selected one strategy after using different strategies at the training-phase and were also able to adapt their strategy fast when necessary. Although technique standardization probably can improve reproducibility, it is advised to retain the freedom for children to choose their own strategy to excel. This is in accordance with the nature of the test and table tennis [3], [7], [8].

Finally, it must be acknowledged that this study only included a small sample size and only a part of this group participated in the retest. This sample size is due to the small number of talented athletes (best 1–5%). These players were considered crucial to include in this study to make fair conclusions for talent identification, consequently reducing the number of athletes. Compared to the subgroup of talented children however, the included sample is believed representative and generalisation should only be done in this selected subgroup. Moreover, although the sample is rather small, we were able to find significant differences between the children from the different training centres, significant association between the test version and competition results and acceptable reproducibility outcomes.

This study can be seen as the first step towards implementation of an eye hand coordination test in a TIDA for table tennis. The results contribute to an evidence-based talent identification program and will help talent development in future. The test version with a table tennis ball at 1 meter distance will be implemented in the NTTA's TIDA as part of talent identification. Further research to obtain normative data and to learn more about the predictive value in longitudinal studies and about reproducibility in a larger sample is essential for correct interpretation of individual test scores of the TIDA [16], [18], [19], [26]. Individual differences due to gender and maturity should also be taken into account [19], [38], [39].

## Supporting Information

Video S1 **Test version table tennis ball – 1 m.**
(MP4)

Video S2 **Test version table tennis ball – 2 m.**
(MP4)

Video S3 **Test version tennis ball – 1 m.**
(MP4)

Video S4 **Test version tennis ball – 2 m.**
(MP4)
